# Quantification of Oxidized and Unsaturated Bile Alcohols in Sea Lamprey Tissues by Ultra-High Performance Liquid Chromatography-Tandem Mass Spectrometry

**DOI:** 10.3390/molecules21091119

**Published:** 2016-08-24

**Authors:** Ke Li, Anne M. Scott, Yu-Wen Chung-Davidson, Ugo Bussy, Trinkal Patel, Zoe E. Middleton, Weiming Li

**Affiliations:** Department of Fisheries and Wildlife, Michigan State University, East Lansing, MI 48824, USA; like4@msu.edu (K.L.); scottan7@msu.edu (A.M.S.); chungyuw@msu.edu (Y.-W.C.-D.); bussy@msu.edu (U.B.); pateltrinkal@gmail.com (T.P.); middle73@msu.edu (Z.E.M.)

**Keywords:** metabolites, sulfated bile alcohol, UPLC-MS/MS, fish

## Abstract

A sensitive and reliable method was developed and validated for the determination of unsaturated bile alcohols in sea lamprey tissues using liquid-liquid extraction and ultra-high performance liquid chromatography-tandem mass spectrometry (UHPLC-MS/MS). The liver, kidney, and intestine samples were extracted with acetonitrile and defatted by *n*-hexane. Gradient UHPLC separation was performed using an Acquity BEH C18 column with a mobile phase of water and methanol containing 20 mM triethylamine. Multiple reaction monitoring modes of precursor-product ion transitions for each analyte was used. This method displayed good linearity, with correlation coefficients greater than 0.99, and was validated. Precision and accuracy (RSD %) were in the range of 0.31%–5.28%, while mean recoveries were between 84.3%–96.3%. With this technique, sea lamprey tissue samples were analyzed for unsaturated bile alcohol analytes. This method is practical and particularly suitable for widespread putative pheromone residue analysis.

## 1. Introduction

**Figure 1:**
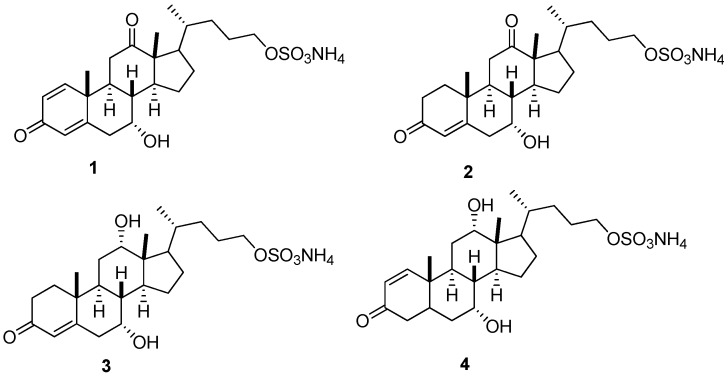
Structures of sulfated BAs analyzed in this study. Compound **1**, 12-keto-1,4-diene 3kPZS; compound **2**, 12-keto-4-ene 3kPZS; compound **3**, 4-ene 3kPZS, and compound **4**, 1-ene 3kPZS.

Sulfated bile salts function as chemical cues that mediate reproduction in sea lamprey, *Petromyzon marinus* [1,2]. Compound 7α, 12α, 24-trihydroxy-5α-cholan-3-one-24-sulfate (3kPZS) is the most abundant bile salt released by sexually-mature male sea lampreys that attracts ovulated females [1,2,3,4]. Analysis of water conditioned with mature male sea lampreys indicated the presence of four additional oxidized, unsaturated compounds [5]. Four unsaturated sulfated bile alcohols (BAs) have been tentatively identified as 7,24-dihydroxy-3,12-diketo-1,4-cholene-24-sulfate (**1**), 7,24-dihydroxy-3,12-diketo-4-cholene-24-sulfate (**2**), 7,12,24-trihydroxy-3-keto-4-cholene-24-sulfate (**3**), and 7,12,24-trihydroxy-3-keto-1-cholene-24-sulfate (**4**) (Figure 1) [5]. Among these compounds, 7,12,24-trihydroxy-3-keto-1-cholene-24-sulfate (**4**) has been demonstrated as a pheromone that attracts ovulated female sea lampreys to spawning nests [5]. To further investigate the physiological mechanism and potential pheromonal functions of these analogs in behavioral experiments, a simple, specific, rapid, and sensitive quantitative method is needed.

The analytical methods for measuring sulfated bile salts are of broad interest for chemists [6,7,8,9,10,11]. A variety of quantitative methods have been reported for sulfated bile acid/salt analysis in biological fluids, such as urine [6,8,9,10,11], bile [10], plasma [10], and stool [7], using gas chromatography (GC) or HPLC in combination with MS. Of these analytical methods, HPLC-MS is more technically advanced. It allows for bile acid profiling without tedious sample purification. Previously reported methods focused on the analysis of 3-sulfated bile salts, which are predominant in humans as detoxification metabolites [12,13]. The lack of direct methods for simultaneous and comprehensive quantification of specific bile salts with C-24 sulfation had led us to develop a LC-MS/MS method to determine and quantify 3kPZS [3], PZS [4], and DkPES [14] to facilitate sea lamprey research. In further investigation of the physiological function of BAs, numerous oxidative and unsaturated BAs have been identified and examined. However, quantitative analytical measurements for highly oxidative and unsaturated BAs are rare. Here we extend our efforts to quantify the oxidized, unsaturated BAs (Figure 1) using UHPLC-MS/MS in sea lamprey tissues. This method was validated and applied to routine analysis in sea lamprey biological research.

## 2. Results and Discussion

### 2.1. Method Development

In order to achieve a good chromatographic separation of all four sulfated BAs analytes with short runtime and low backpressure, three analytical columns were compared, namely, Acquity™ UPLC BEH (Waters Corporation, Milford, MA, USA) C18 column (50 mm × 2.1 mm, 1.7 µm particle size), BEH C18 column C18 (100 mm × 2.1 mm, 1.7 µm particle size), and HSS T3 C18 column, (15 mm × 2.1 mm, 1.8 µm particle size). As expected, the BEH C18 column showed improved peak resolution since it incorporates trifunctional ligand binding chemistries on 1.7 µm BEH particles and utilizes new endcapping processes resulting in increased stability over a wide pH range [15], lower resistance to mass transfer, and ultimately higher resolution [16,17]. The separation performance on BEH (100 mm and 50 mm) columns have been evaluated. All analytes were resolved most rapidly using a 50 mm column (data not shown). Therefore, the BEH C18 column (50 mm × 2.1 mm, 1.7 µm particle size) was selected for use in the following work.

The separation of sulfated BAs (**1**–**4**) were achieved by a gradient consisting of 20 mM triethylamine (TEA) in water (solvent A) and 20 mM TEA in methanol (solvent B). The gradient with TEA has been widely used for the quantification of sulfated BAs in biological fluids, such as plasma, and other tissues [3,4]. The primary impediment for baseline separation and well-shaped peaks of bile alcohol/acid was due to the hydroxyl groups on the steroid backbone and the anionic groups located at the end of the side chain. Previous studies used ammonium acetate and formic acid as additives to the mobile phase to prevent the tailing and distortion of peaks in the chromatogram [18,19,20,21]. In this study, four targeted sulfated bile acids displayed sufficient resolution with TEA buffer (20 mM) in mobile phase, which had also been applied in our previous report [3].

**Figure 2:**
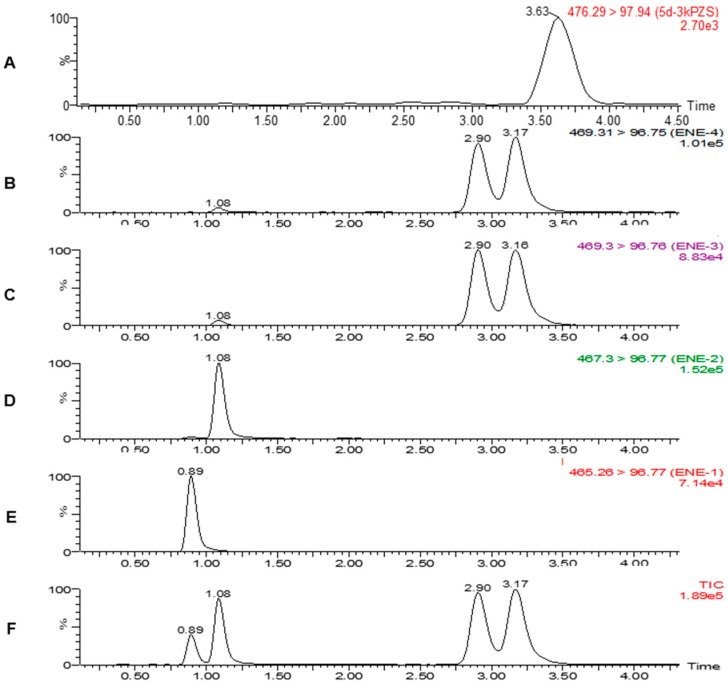
Chromatograms of (**A**) [^2^H_5_]-3kPZS, with transition 476 > 97; (**B**) 1-ene 3kPZS with transition 469 > 97; (**C**) 4-ene 3kPZS with transition 469 > 97; (**D**) 12-keto-4-ene 3kPZS with transition 467 > 97; (**E**) 12-keto-1,4-diene 3kPZS with transition 465 > 97; and (**F**) four targeted sulfated BAs; by LC-MS/MS. Analyte standards: 100 ng/mL each, internal standard 10 ng/mL.

Bile acid compounds are commonly detected using ESI in the negative-ion mode because of the carboxylate group in their molecular structure [21,22,23]. ESI-MS/MS using low-energy CID has been used in the quantification of BAs in biological fluids [24], human serum [24,25,26], human urine [27], and rat serum [28]. Multiple reaction monitoring (MRM) mode with specific detection of precursor/product ion pairs pinpoints and accurately measures each analyte (Table 1). In addition, ESI-MS/MS on negative-ion mode at low collision-energy performed on sulfated bile alcohol results in common fragment ion at *m*/*z* 96 (HSO_4_^−^), which was usually used for determination of sulfated BAs [24,26]. Therefore, it was critical to obtain sufficient separation of the four target analytes through chromatography since they would generate similar fragmentation (Table 1) and, consequently, nondistinguishable in mass spectrometry even with MRM mode. Separation of the four sulfated bile alcohol standards was completed in a single run of 7 min (Figure 2) including column equilibration. The gradient was shown in the Experimental section. The 7 min run time including column equilibrium is an improvement for bio-sample analysis compared to 12 min in the bile acid quantification method developed for lake char feces [19].

### 2.2. Calibration and Method Validation

The calibration curves were linear in the range from 0.1 to 1000 ng/mL for 12-keto-1,4-diene 3kPZS and 12-keto-4-ene 3kPZS, and 0.2–1000 ng/mL for 4-ene 3kPZS and 1-ene 3kPZS. The correlation coefficients for the standard calibration curves for these four analytes were higher than 0.992, indicating method linearity (Table 1).

To ensure method reliability and reproducibility for BA analysis, intra- and inter-day accuracy and precision were determined using three QC concentrations distributed throughout the calibration range for each analyte in tissue homogenate matrix. Intra- and inter-day precision and accuracy data are summarized in Table 2. For precision of BAs, intra-day variation (*n* = 6) ranged from 0.31% to 4.03%, and inter-day from 1.52% to 5.28%. Intra-day accuracy (*n* = 6) ranged from 95.5% to 99.3%, and inter-day from 95.7% to 101.1%. The precision and accuracy for all analytes are greater than 85% and, therefore, are within the interval set by the FDA concerning the validation of bioanalytical methods. The limit of detection for each compound was determined at the lowest concentration showing a signal to noise ratio (*S*/*N*) higher or equal to three and ranged from 0.1 to 0.2 ng/mL (Table 1). Furthermore, the stability of stock solutions under storage conditions and the stability of extracted biological samples in the autosampler were tested. These analytes were stable for at least 14 days in –20 °C freezer, and 96 hrs in 4 °C autosampler (data not shown).

LOQ was defined as the lowest concentration showing a signal with *S*/*N* ≥ 10. This concentration also satisfied the following conditions: a coefficient of variation below 20% and accuracy between 80% and 120% (*n* = 6). For these analytes, LOQ ranged from 0.33 ng/mL to 0.66 ng/mL. Compared to similar sulfated bile acid quantitative analysis, this method exhibits lower LOD and LOQ, and higher accuracy and precision [29,30].

### 2.3. Matrix Effects

The results of the quantitative matrix effect tests (*n* = 5) are shown in Table 3. Matrix effect is the only factor that was taken into account since the analytes were added to the matrix after extraction and compared to the same amount of analytes in pure solvents. The matrix effects ranged from 85.9% ± 1.6% to 99.3% ± 3.2% (Table 3) in liver, from 85.6% ± 1.9% to 98.6% ± 2.3% in kidney, and from 85.9% ± 1.8% to 95.6% ± 1.9% in intestine at concentrations of 10, 100, and 500 ng/g, whereas the matrix effect for IS (100 ng/mL) was 90.5% ± 2.2%. Therefore, negligible matrix effects were observed.

### 2.4. Quantitative Analysis of Sulfated BAs in Tissues

**Figure 3:**
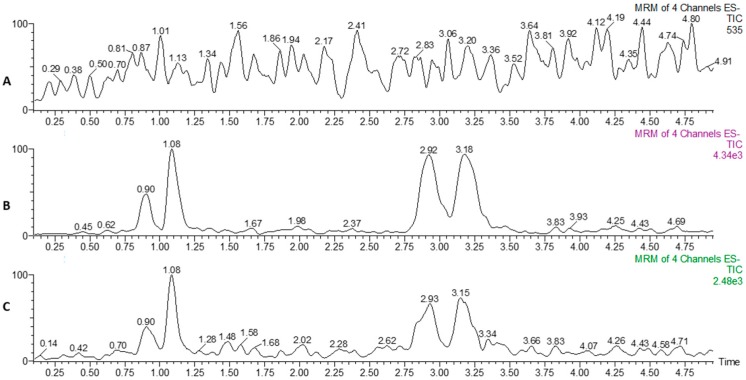
Representative chromatograms of a sample extracted from kidney (**A**); liver (**B**); and intestine (**C**).

This validated method was applied to measure analytes in tissue samples from the sea lamprey (Figure 3). The concentrations of each compound in sea lamprey liver, kidney, and intestine are summarized in Table 4. The concentrations of 12-keto-1,4-diene 3kPZS and 12-keto-4-ene 3kPZS in liver, kidney, and intestine were undetectable by the developed method. The analytes 4-ene 3kPZS and 1-ene 3kPZS showed trace amounts in liver and intestine extracts. The concentrations of the analytes 4-ene 3kPZS and 1-ene 3kPZS were 0.81 and 0.92 ng/g in liver extract, 0.85 and 0.74 ng/g in intestine extract, respectively. These results suggest that 12-keto-1,4-diene 3kPZS, 12-keto-4-ene 3kPZS, 4-ene 3kPZS, and 1-ene 3kPZS are minute metabolites in the liver and intestine of sea lamprey.

## 3. Experimental Section

### 3.1. Chemicals and Reagents

Methanol (Sigma-Aldrich, St. Louis, MO, USA), hexane (Sigma-Aldrich), ethanol (Sigma-Aldrich), and triethylamine (TEA, Sigma-Aldrich) were HPLC grade. De-ionized water was prepared using a Milli-Q system (Millipore, Billerica, MA, USA).

7,24-dihydroxy-3,12-diketo-1,4-choladiene-24-sulfate (12-keto-1,4-diene 3kPZS), 7,24-dihydroxy-3,12-diketo-4-cholene-24-sulfate (12-keto-4-ene 3kPZS), 7,12,24-trihydroxy-3-keto-4-cholene-24-sulfate (4-ene 3kPZS), 7,12,24-trihydroxy-3-keto-1-cholene-24-sulfate (1-ene 3kPZS), and deuterated 3-keto-petromyzonol sulfate ([^2^H_5_]-3kPZS) were custom-synthesized by Bridge Organics Co. (Vicksburg, MI, USA; Figure 1). The reported purities were above 95%.

### 3.2. Preparation of Calibration Standards, Quality Control, and Internal Standard Solutions

The four sulfated bile alcohol reference standards (Figure 1) were separately dissolved in methanol as individual stock solutions (1 mg/mL). Stock solutions were combined and then serially diluted to produce standard solutions for a calibration curve (0.05, 0.1, 0.2, 0.5, 1.0, 2.0, 5.0, 10.0, 20.0, 50.0, 100.0, 200.0, 500.0, 1000.0, 2000.0, 5000.0 ng/mL for each compound). Quality control (QC) samples were prepared at three concentration levels as higher, middle, and lower limit of quantification, and abbreviated as HQC, MQC, and LQC, respectively, based on the dynamic ranges of the analytes (Table 1). [^2^H_5_]-3kPZS was used as the internal standard (IS; Bridge Organics Co. Vicksburg, MI, USA), and dissolved in methanol to produce a 10 ng/mL IS solution. All stock solutions were sealed and stored at −20 °C until use.

### 3.3. Quantitative Conditions by UHPLC-MS/MS

The LC-MS/MS system consisted of a Waters ACQUITY H-Class UPLC™ system connected to a Waters Xevo TQ-S triple quadrupole mass spectrometer (Waters Corp., Milford, MA, USA). The mobile phase consisted of water (containing 20 mM TEA) as (A) and methanol (containing 20 mM TEA) as (B). A Waters BEH C18 column (2.1 mm × 50 mm, 1.7 µm particle size) coupled with an Acquity UPLC™ column in-line filter kit (0.2 µm filter) was used. Separation was achieved using the following gradient program at a flow rate of 250 µL/min for 7 min at 35 °C: 70% A for 1 min, decreased to 20% A from 1 to 4 min, decreased to 0% A from 4 min to 4.01 min, and then maintained at 0% A from 4.01 to 5.00 min, increased to 70% A from 5.0 to 5.01 min, and stayed at 70% A to 7 min for column equilibrium. The injection volume was 10 µL. The retention times for analytes 4-ene 3kPZS and 1-ene 3kPZS were determined by individual injection of each analyte. These sulfated BAs were detected by MRM mode and processed by Masslynx 4.1 software (Waters Corporation, Milford, MA, USA). The transition for each analyte was listed in Table 1.

The UHPLC effluent was introduced into the mass spectrometer with electrospray ionization in the negative mode. The ESI-MS/MS parameters were set as follows: capillary voltage, 2.60 kV; extractor voltage, 5 V; source temperature, 150 °C; desolvation temperature, 500 °C; desolvation gas flow, 800 L/h (N_2_, 99.9% purity). Argon (99.9999% purity) was introduced as the collision gas into the collision cell at a flow rate of 0.15 mL/min. Data were collected in centroid mode with a scan range of 50–1000 *m*/*z*. MRM transitions and related parameters are listed in Table 1. The dwell time established for each transition was 0.2 s, and interscan delay was set at 20 ms. The internal standard (1.0 μg/mL, 10 μL) was added to each sample. Data acquisition was carried out by Masslynx 4.1 software and processed by TargetLynx (Waters Corp.).

### 3.4. Tissue Sample Preparation, Extraction, and Quantification

One gram of homogenized sample was weighed into 15 mL polypropylene tube. The sample was spiked with the internal standard and incubated for 15 min. Acetonitrile (5 mL) was added to the sample, vortexed for 1 min, shaken at 300 rpm for 10 min, and followed by centrifugation at 10,000 rpm for 10 min. The supernatant was transferred to a clean 15 mL polypropylene tube. Hexane (5 mL) was added into the extracted supernatant. After being vortexed for 1 min, the mixture was centrifuged at 10,000 rpm for 10 min. The upper hexane layer was discarded. The lower acetonitrile layer was concentrated by a freeze dryer, reconstituted in 100 μL of the starting gradient of the mobile phase, and transferred into an auto sampler vial. Each sample (10 μL) was injected by the autosampler. The concentration of each sample was measured by plotting the ratio of the peak area to that of the internal standard against a calibration curve constructed by standard analyte with internal standard. Data were processed by Masslynx 4.1 software, TargetLynx module.

### 3.5. Method Validation

The UPLC-MS/MS method was validated for specificity, linearity, limit of detection (LOD), lower limit of quantification (LLOQ), intra-day and inter-day accuracy and precision, short-term (bench-top and freeze/thaw) stability, long-term frozen storage stability, matrix effect, and extraction recovery.

Specificity was evaluated by a chromatographic peak area comparison between the blank sample and the standard sample. The peak area at the expected retention time of each analyte in blank samples should be less than 20% of the average peak area in the LLOQ samples [31].

Standard solutions were prepared for each batch of sample analysis, as described in Section 3.2. A calibration curve was constructed by plotting the ratio of the peak area of the measured analyte to that of the internal standard in the Y-axis versus the theoretical concentration of the analyte in the X-axis. Linear least squares regression analysis with 1/χ^2^ weighting was performed, and the slope, intercept, and correlation coefficient (r^2^) of the calibration curve were calculated (Masslynx 4.1 software, TargetLynx module).

The LLOQ of the assay was defined as the lowest concentration of the standard curve that could be quantified (LLOQ, signal-to-noise ratio *S*/*N* ≥ 10). The LOD was defined as the amount that could be detected (LOD, *S*/*N* ≥ 3). The LLOQ and LOD of this study have been measured with the stock solution.

The accuracy and precision of the established method were evaluated by QC samples at low, medium, and high concentrations in a mixture of liver, kidney, and intestine tissue. Three validation batches, each containing six replicates of QC samples at low, medium, and high concentration levels were assayed to assess the precision and accuracy of the method on five consecutive validation days. Precision of the assay is expressed as a % RSD of the measured concentration in each low, medium, and high QC samples. Accuracy of the assay is expressed as a percentage of deviation/bias of the measured concentration from the nominal concentration. The intra-day and inter-day precision and accuracy should not exceed 15% [31].

The stability of the analytes was determined by storing low, medium, and high QC samples under various conditions. To evaluate bench-top stability of the analytes, the QC samples were stored at ambient temperature (23–25 °C) for 1, 2, 3, and 4 h. To determine the post-preparative stability during UPLC-MS/MS analysis, the QC samples were kept at 4 °C in the autosampler for 3, 6, 12, and 24 h. To evaluate long-term stability, the prepared QC samples were kept at −20 °C for seven and 14 days.

The extraction recoveries of the analytes at three QC levels were evaluated by determining the ratios of the peak area of the analytes in the post-extraction spiked samples to that of pre-extraction spiked samples.

To assess matrix effects in a quantitative manner, six samples were extracted using the procedures described in Section 3.4. After extraction, three samples were spiked with defined low, medium, and high amounts of standard stock solutions (10, 100, and 500 ng/ml, respectively), while the remaining three samples were kept as the blank. Simultaneously, identical amounts of standard stock solutions and internal standards were pipetted into three clean vials. All samples were then evaporated and dissolved in the mobile phase solution according to sample preparation and extraction. After correcting the spiked samples by subtracting endogenous amounts of the respective analyte, quantitative matrix effects were assessed by dividing the peak area of spiked samples (with matrix) to the peak area of samples containing standard solutions (without matrix).

### 3.6. Data Analysis and Statistics

TargetLynx 4.1 software was used to generate “quantification tables” comprising RT, signal area values, and concentration for each variable in each sample. Linear relationship calculations between signal areas and concentrations were acquired by weighted least squares regression. The results were presented as mean ± standard deviations.

## 4. Conclusions

In this study, we developed a new UHPLC-MS/MS method for simultaneous quantitative analysis of four sulfated BAs in biological samples. The method achieved a sensitive, precise, and accurate determination of metabolite targets in liver, kidney, and intestine of the sea lamprey. This method will be useful to study the metabolites released by fish, and will support future studies on the role of sulfated BAs as chemical cues.

## Figures and Tables

**Table 1:** Optimized UHPLC-MS/MS parameters for each analyte.

Compounds	[M − H]^−^ *m*/*z*	MRM *m*/*z*	CV (V)	CE (eV)	RT (min)	LR	R^2^	LOD (ng/g)	LOQ (ng/g)
**1**	465.2	465.2 > 96.7	49	40	0.89	0.1–1000	0.9918	0.10	0.33
**2**	467.3	467.3 > 96.7	23	40	1.08	0.1–1000	0.9924	0.10	0.33
**3**	469.3	469.3 > 96.7	36	40	2.90	0.2–1000	0.9973	0.20	0.66
**4**	469.3	469.3 > 96.7	23	40	3.16	0.2–1000	0.9952	0.20	0.66
[^2^H_5_]-3kPZS	476.3	476.3 > 96.7	100	23	3.05	0.5–1000	0.9986	0.30	0.99

*m*/*z* corresponds to [M − H]^−^, MRM, multiple reaction monitoring; CV, cone voltage; CE, collision energy; RT, retention time. RTs were derived from Figure 2. LR, linear range; R^2^, correlation coefficient; LOD, limit of detection; LOQ, limit of quantitation.

**Table 2:** Intra-day and inter-day accuracy and precision of this LC/MS/MS method.

Analyte	NC (ng/g)	Intra-Day (*n* = 6)				Inter-Day (*n* = 6)	
		MC (Mean ± SD, ng/mL)	Accuracy (DEV %)	Precision (RSD %)	MC (Mean ± SD, ng/mL)	Accuracy (DEV %)	Precision (RSD %)
**1**	2.5	2.4 ± 0.03	96.9	1.24	2.4 ± 0.11	95.2	4.80
	25	24.1 ± 0.50	96.5	2.07	24.1 ± 1.22	96.4	5.05
	250	248.2 ± 1.18	99.3	0.48	245.8 ± 5.84	98.3	2.38
**2**	2.5	2.4 ± 0.03	95.5	1.22	2.5 ± 0.10	101.1	4.29
	25	24.1 ± 0.72	96.3	2.99	25.0 ± 1.03	100.1	4.12
	250	243.6 ± 5.10	97.4	2.09	247.3 ± 3.77	98.9	1.52
**3**	2.5	2.4 ± 0.05	97.1	1.96	2.4 ± 0.04	95.7	1.81
	25	24.1 ± 0.89	96.4	3.70	25.2 ± 1.06	100.8	4.21
	250	248.3 ± 2.03	99.3	0.82	247.3 ± 4.25	98.9	1.72
**4**	2.5	2.41 ± 0.04	96.3	1.67	2.4 ± 0.04	97.03	1.87
	25	24.6 ± 0.99	98.3	4.03	25.2 ± 0.93	100.6	3.69
	250	247.5 ± 0.76	99.0	0.31	250.3 ± 13.21	100.1	5.28

Note: NC, nominal concentration; MC, measured concentration; SD, standard deviation, DEV, deviation; RSD, relative standard deviation in percentage.

**Table 3:** Mean extraction recoveries and matrix effect of the analytes in liver, kidney, and intestine extract (*n* = 5). Percentage SDs are in parentheses.

Matrix	Analyte	Mean Extraction Recovery (%)			Matrix Effect (%)		
		Low	Medium	High	Low	Medium	High
Liver	**1**	96.3 (2.3)	95.6 (3.1)	92.9 (1.5)	97.3 (4.1)	99.3 (3.2)	96.3 (2.9)
	**2**	92.3 (1.8)	95.2 (2.0)	91.3 (3.2)	90.3 (3.2)	92.3 (2.9)	92.6 (2.1)
	**3**	87.9 (3.1)	89.6 (2.7)	88.6 (3.4)	89.6 (3.5)	87.9 (3.2)	88.6 (2.8)
	**4**	92.3 (2.6)	86.3 (2.5)	84.3 (2.7)	85.9 (1.6)	95.6 (2.7)	89.6 (2.2)
Kidney	**1**	96.3 (2.5)	95.8 (1.4)	95.6 (3.2)	98.6 (2.3)	96.3 (4.2)	95.8 (4.9)
	**2**	95.3 (2.4)	95.6 (2.5)	94.3 (4.5)	88.9 (3.1)	87.6 (2.2)	89.6 (1.8)
	**3**	86.9 (3.0)	89.6 (1.9)	92.3 (3.1)	87.9 (3.8)	86.9 (2.7)	89.6 (3.6)
	**4**	91.2 (2.0)	89.6 (1.7)	93.2 (2.9)	92.3 (1.1)	85.6 (1.9)	86.9 (1.7)
Intestine	**1**	96.3 (3.3)	95.3 (2.7)	94.2 (2.5)	95.3 (3.6)	94.3 (2.8)	95.6 (1.9)
	**2**	94.2 (2.9)	95.3 (3.1)	92.3 (2.9)	93.6 (4.0)	92.7 (3.8)	93.5 (2.5)
	**3**	86.3 (3.2)	87.5 (4.1)	89.6 (1.9)	87.9 (3.5)	85.9 (1.8)	89.6 (1.7)
	**4**	92.5 (3.4)	95.6 (2.9)	91.5 (3.2)	95.2 (1.3)	93.2 (2.7)	94.3 (1.6)

**Table 4:** Concentration of each analyte in sea lamprey liver, kidney, and intestine.

Sample	Concentration (ng/g)			
	1	2	3	4
Kidney	N.D	N.D	N.D	N.D
Intestine	N.D	N.D	0.85	0.74
Liver extract	N.D	N.D	0.81	0.92

N.D not detected.
